# Post-Thaw Day 5 Blastocyst Culture Time Prior to Transfer Does Not Affect Assisted Reproduction Technology (ART) Outcomes in Frozen-Thawed Embryo Transfer Cycles

**DOI:** 10.3390/jcm11247444

**Published:** 2022-12-15

**Authors:** Marta Ciaffaglione, Marco Reschini, Martina Balli, Cristina Guarneri, Maria Carla Palermo, Monica Pinna, Valerio Pisaturo, Edgardo Somigliana, Alessio Paffoni, Paola Vigano’

**Affiliations:** 1 Infertility Unit, Fondazione IRCCS Ca’ Granda Ospedale Maggiore Policlinico, 20122 Milan, Italy; 2 Department of Clinical Sciences and Community Health, Università degli Studi di Milano, 20122 Milan, Italy; 3 Infertility Unit, ASST Lariana, 22063 Cantù (Como), Italy

**Keywords:** infertility, IVF, reproduction

## Abstract

The frozen embryo transfer (FET) technique has been progressively used more worldwide due to improved culture conditions, as well as enhanced survival rates after vitrification. However, little is known about the effect of the post-thaw blastocyst culture duration prior to transfer on live birth rate in FET cycles. In this retrospective observational study, we evaluated the influence of two distinct post-thaw blastocyst culture spans (2–4 h versus 20–22 h) on clinical pregnancy and live birth rate. A total of *n* = 1927 frozen–warmed cycles were included in the analysis. Among those, *n* = 885 warmed blastocysts were cultured for 2–4 h, and *n* = 1029 were kept in culture for 20–22 h prior to transfer; the remaining blastocysts did not survive the warming protocol. We observed no significant differences in live birth and clinical pregnancy rates between the two groups. The blastocyst morphological evaluation at transfer improved following the longer culture time. No differences between the two groups were found also for gestational and neonatal outcomes. This work shows that different post-thaw embryo culture timings do not negatively impact pregnancy outcomes. Overall, these results are important in the context of the embryological laboratory in order to better organize the workflow and avoid unnecessary timing-related workload.

## 1. Introduction

In recent decades, assisted reproduction technology (ART) treatments have been enormously shaped by the advancement of cryopreservation methods, leading to widespread use of the frozen embryo transfer (FET) strategy [1]. Frozen embryo transfer reduces the risk of ovarian hyperstimulation syndrome (OHSS) without facing adverse effects due to the supraphysiological hormonal levels on endometrial receptivity [2]. Moreover, with FET, elective single-embryo transfer has become globally acknowledged, resulting in the reduction in the rates of twins and multiple pregnancies [1,3]. Usually, according to the FET strategy, after warming, embryos can be transferred into the uterus either after a short culture duration of 2 to 5 h [4,5,6] or after a longer culture duration, defined as a minimum of 16 h of post-thawing culture time [7,8]. Different embryo culture durations post-thawing can be translated into better laboratory organization, as well as more personalized workflow, where embryos can be warmed a day prior to the transfer. A recent meta-analysis performed by Sordia-Hernandez et al. reported no statistically significant differences in terms of implantation rate, clinical pregnancy, and live birth rates between short and long embryo culture after warming [7]. However, of the five included studies, one study considered exclusively transfers of day 5 embryos, while other studies included transfers of day 3 embryos. The meta-analysis is thus of limited value in addressing the effect of the post-warming culture period between thawing and transfer of cryopreserved blastocysts. To the best of our knowledge, only five studies have been published so far on the impact of the duration of post-warming culture on results of blastocyst transfers [9,10,11]. According to Guerif et al., a long post-thaw embryo culture may be associated with improved biological outcomes in terms of blastocoel expansion and development, as well as with significantly increased implantation and clinical pregnancy rates compared to a short culture period [9]. Herbemont and colleagues demonstrated that a long, post-warming embryo culture duration positively affected blastocyst re-expansion and hatching rates compared to the short-culture group. Nonetheless, both implantation and live birth rate were not influenced by the distinct embryo culture duration after warming [10]. Similar findings were obtained by Hwang and coworkers [11]. Haas and coworkers could not provide comparative results between the two strategies [12]. Finally, very recently, Ji et al., considering 3.901 frozen-thawed blastocyst transfer cycles, showed no statistical differences in live birth and implantation rates following transfers of day 5 blastocysts cultured for a short or a long period post-thawing [13]. Conversely, following day 6 blastocyst transfer, patients in the long-culture group had significantly lower outcomes than those in the short-culture group. Overall, although some studies have tried to unravel which is the best blastocyst culture duration period post-warming during FET cycles (Table 1), the answer to this question remains unclear. Indeed, the studies are few, and most of them are defined by a small sample size.

Here, we demonstrate the influence of a short (2–4 h) and long (20–22 h) post-thaw culture period on day 5 blastocysts prior to transfer. Specifically, we confirm that FET can be efficiently carried out after either a short or long day 5 blastocyst culture duration, followed by similar results in terms of clinical pregnancy and live birth rates.

## 2. Materials and Methods

### 2.1. Study Design, Size, and Duration

This retrospective study was conducted at the Infertility Center—Fondazione IRCCS Ca’ Granda Ospedale Maggiore Policlinico in Milan between 2014 and 2021. We included couples with IVF indications who underwent frozen–thawed blastocyst transfer either after a freeze-all strategy or for supernumerary embryos. Only transfer of single blastocysts vitrified at day 5 and warmed were evaluated. The choice on the timing of blastocyst culture after thawing was based essentially on the workload and organizational preferences of the embryologists and not on clinical or embryological parameters. We primarily compared the live birth rate of patients undergoing FET after a short blastocyst culture time (2–4 h) prior to transfer to that of patients in whom thawed blastocysts were transferred after a long embryo culture time (20–22 h). The secondary outcomes of the study were clinical pregnancy and miscarriage rates. Clinical pregnancy rate was defined by the presence of at least one intrauterine gestational sac, assessed by the sonographic documentation, divided by the number of embryo transfer cycles for each group, while miscarriage rate was defined as a spontaneous loss of pregnancy before the 12th week of gestation divided by the number of clinical pregnancies for each group. In accordance with Italian law, all pregnant women were followed up with, and pregnancy and neonatal data, including rates of hypertension, gestational diabetes, placenta previa, preterm birth (birth of an infant before 37 completed weeks of gestation), and low birth weight (LBW—defined as a birth weight of fewer than 2500 g) were precisely collected. The local Ethics Committee Milan Area 2 approved the research protocol (approval no. 28_2022, 11 January 2022).

### 2.2. Controlled Ovarian Stimulation and Embryo Culture

Controlled ovarian stimulation was performed after assessing age, day 3 serum FSH, serum anti-Mullerian hormone (AMH), and antral follicle count (AFC) in order to define the proper gonadotropin type to be used, as well as the starting dose [14]. All patients were treated with a GnRH antagonist protocol. Briefly, both the initial dose of recombinant or urinary FSH and dose adjustments during treatment were chosen on a case-by-case basis according to patients’ characteristics. A daily dose of 0.25 mg of GnRH antagonist was started upon the visualization of a dominant follicle of 13–14 mm. During the stimulation, women underwent several transvaginal ultrasounds, evaluating the ovarian response, and, possibly, adjustment of the gonadotropin dosage. When three or more leading follicles had reached a diameter > 17 mm, the triggering of ovulation was performed with 250 μg of high recombinant human chorionic gonadotrophin, or 0.2 mL GnRH agonist in case of risk of ovarian hyperstimulation syndrome. Oocyte retrieval was transvaginally performed 36 h later [15]. Intracytoplasmic sperm injection (ICSI) and conventional IVF were standardly conveyed. A fertilization check for two pronuclei took place 16–18 h later. Subsequently, embryos were group cultured in sequential media (G-1™ PLUS-Vitrolife, Stockholm, Sweden) up to developmental day 3 [16]. Embryo quality was checked for number and size of blastomeres and degree of fragmentation, as well as for multinucleation [17,18]. Embryos were defined as good with <10% fragmentation, stage-specific cell size, and no multinucleation; fair with 10–25% fragmentation, stage-specific cell size for majority of cells, and no evidence of multinucleation; poor with severe fragmentation (>25%), cell size not stage-specific, and evidence of multinucleation. Top quality embryos were defined by a stage-appropriate number of evenly sized blastomeres, complete absence of multinucleation, and less than 10% fragmentation [19].

In our laboratory, a shared embryo quality control, as well as other subjective assessments among the embryologists, is undertaken with a biannual frequency in order to standardize the evaluations. In any case, the quality of the embryos has been generally evaluated by the same embryologist (head of the lab) who is assigned to this specific task.

### 2.3. Blastocyst Evaluation and Cryopreservation

After 5 days of culture, blastocyst evaluation was performed according to the Istanbul Consensus [19]. In line, a score from 1 to 4 was assigned based on the blastocyst degree of expansion and hatching status (1 as early expansion, 2 as blastocysts, 3 as fully expanded, 4 as hatched/hatching). Hatching was defined as the obvious emergence of the trophectoderm with enclosed blastocoel through a thinning zona pellucida. The inner cell mass was scored as follows: 1—prominent, easily discernible, with many cells that are compacted and tightly adhered together; 2—easily discernible, with many cells that are loosely grouped together; 3—difficult to discern, with few cells. The trophectoderm was scored as follows: 1—many cells forming a cohesive epithelium; 2—few cells forming a loose epithelium; 3—very few cells. ‘Top–good quality’ blastocysts were defined as advanced blastocysts (type 3 expanded blastocyst and type 4 hatched/hatching blastocyst) with an inner cell mass scored 1 or 2 and a multicellular trophectoderm (scored 1 or 2). Blastocysts were vitrified after artificial shrinkage by a single laser shot on the trophoblast cells [20,21], according to the Kuwayama method [22]. The vitrification was performed using a vitrification kit (Kitazato, Tokyo, Japan).

### 2.4. Frozen–Thawed Embryo Transfer

Women with frozen blastocysts were scheduled for a natural cycle if they reported having regular menstrual cycles and a mean cycle length between 24 and 35 days. Eligible women were monitored with sequential transvaginal ultrasounds starting from day 6–9 of the cycle, and a luteinizing hormone (LH) surge assessment was achieved by monitoring urinary sticks for an LH surge three times per day (7 a.m., 3 p.m., and 10 p.m.) until the leading follicle reached 16 mm. Four days after the LH surge (+3 from presumed ovulation), women underwent a transvaginal ultrasound and serum progesterone assessment. Embryo transfer was performed 6 days after LH surge (+5 from presumed ovulation). No luteal phase support was given. Hormone replacement treatment (HRT) was prescribed if women had irregular menstrual cycles or if the monitoring of the natural cycle failed. Specifically, they received daily 4 mg of estradiol valerate orally from day 2 of the cycle that was increased to 6 mg after 6 days. The dose was further increased to 8 mg if the thickness of the endometrium did not reach 7 mm. Progesterone was then introduced progressively, reaching the plateau (400 mg vaginally twice a day) after two days (200 mg once the first day, 200 mg twice a day the second day, and 400 mg twice a day from the following day onwards) [23]. Single-embryo transfer was performed at day 5 from initiation of progesterone. According to the policy of our unit, all women underwent single-embryo transfer.

Blastocysts were warmed using the thawing kit (Kitazato, Japan) [22] and cultured in G-2™ PLUS (Vitrolife, Sweden) [16] before transfer. Blastocysts were either thawed on the day of embryo transfer and cultured for 2–4 h before transfer (short culture) or thawed in the afternoon of the day before embryo transfer and cultured overnight for 20–22 h before embryo transfer (long culture). The blastocyst quality was evaluated at the time of transfer.

### 2.5. Statistical Analysis

Data were analyzed using the SPSS software 27.0 (IBM Corp, Armonk, NY, USA). The Fisher exact test, chi-squared test and Wilcoxon’s test were used as appropriate, and *p* values below 0.05 were considered statistically significant. A binomial distribution model was used to calculate the 95% confidence interval (95% CI) of proportions. A logistic regression model was used to calculate the adjusted odds ratio (OR) of pregnancy and live birth. Variables included in the model were age and number of oocytes retrieved. The sample size was calculated in order to demonstrate that the transfer of frozen–thawed blastocysts after a long embryo culture (20–22 h) could lead to a relative reduction of the live birth rate probability ≤ 20% (from 30 to 24%). Setting type I and II errors at 0.05 and 0.20, respectively, a sample size of *n* = 860 blastocysts for each group was deemed appropriate.

## 3. Results

Between 2014 and 2021, we analyzed a total of *n* = 1927 day 5 blastocysts FET cycles, of which *n* = 885 (46%; 95%CI: 44–48%) warmed blastocysts were cultured for 2–4 h (short culture), and *n* = 1029 (53%; 95%CI: 51–56%) were kept in culture for 20–22 h (long culture) prior to transfer; the remaining 13 blastocysts (1%; 95%CI:0–1%) did not survive the warming protocol.

The basal characteristics of the patients who underwent FET cycles with the short and long blastocyst culture were similar (Table 2). The median [interquartile range] number of day 3 embryos from which blastocysts were derived was also similar in both groups (7 [5–8] and 6 [5–8], respectively).

**Figure 1:**
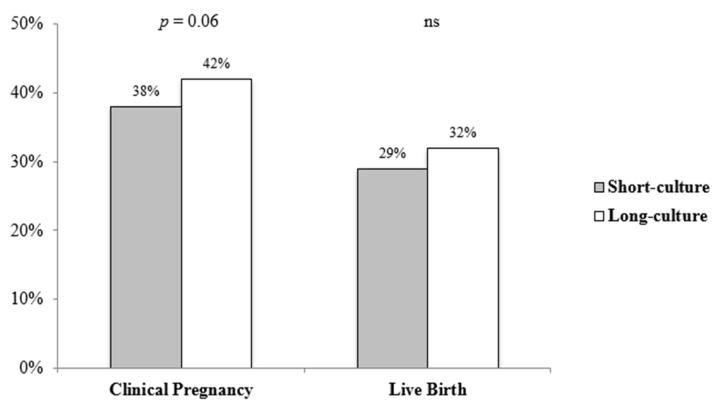
Clinical pregnancy and live birth rate indications of the patients who underwent FET cycles divided according to the timing of blastocyst culture before transfer (short, 2–4 h and long, 20–22 h).

Among the 13 blastocysts that did not survive warming, *n* = 5 (0.5%) belonged to the short-culture group and *n* = 8 (0.7%) to the long-culture group. No statistically significant differences were found between the short- and long-culture groups in live birth rate after FET (29% versus 32%, *p* = 0.13, respectively), suggesting that the distinct culture duration prior to blastocyst transfer does not negatively impact the main ART goal (Figure 1). Furthermore, we observed a trend of a lower clinical pregnancy rate following the transfer of blastocysts that were shortly cultured prior to transfer compared to the ones that were cultured overnight (38% versus 42%, respectively, *p* = 0.06) (Figure 1). The OR of live birth following the long culture, adjusted for age and number of oocytes retrieved, resulted in 1.17 [0.97–1.42; *p* = 0.11]. The OR of the clinical pregnancy rate following the long culture, adjusted for age and number of oocytes retrieved, resulted in 1.20 [0.99–1.44; *p* = 0.06].

We have also assessed blastocyst morphological features. At vitrification, 28 (3%) and 66 (6%) of blastocysts were early blastocysts (grade 1), 335 (38%) and 549 (54%) were blastocysts (grade 2), 515 (58%) and 412 (40%) were fully expanded (grade 3), and 7 (1%) and 2 (<1%) were hatched (grade 4) in the short- and long-culture groups, respectively. Even if blastocysts, that were transferred following a short culture post-warming, displayed a significantly superior morphology at the vitrification moment, those that were warmed the day before (i.e., cultured overnight for 20–22 h) displayed a significantly improved morphology at transfer (Table 3).

A higher number of less-expanded blastocysts at vitrification were cultured for a longer period. On the other hand, independent from the original expansion, blastocysts seemed to benefit more from a longer culture. In this group, trends toward a higher live birth rate and a significantly higher clinical pregnancy rate were observed for blastocysts that, at vitrification, were at grades 1–2 or 3–4 of expansion, respectively.

To gather more knowledge on the influence of blastocyst culture duration on the most relevant ART outcomes, we subsequently assessed pregnancy and neonatal outcomes after transfers of single warmed blastocysts vitrified at day 5. Rates of spontaneous abortion, ectopic pregnancy, intrauterine fetal death, gestational diabetes, hypertension, placenta previa, preterm labor, low birth weight babies, per blastocyst transferred, were found to be similar between the short and long culture group (Table 4).

## 4. Discussion

Overall, our findings demonstrate that different culture timings after warming of day 5 blastocysts have similar ART clinical outcomes. Specifically, in terms of clinical pregnancy and live birth, no differences were noticed between the short-(2–4 h) and long-culture (20–22 h) groups. Therefore, both culture approaches may be efficiently applied, and the decision would be based on the laboratory workflow.

The use of frozen embryo transfers has steadily increased over the past decade, and this approach to ART is now practiced worldwide [24]. This strategy is generally associated with the single-embryo transfer, mostly at the blastocyst stage [3]. Therefore, in the daily practice of ART centers, blastocyst thawing and transfer is becoming one of the most performed procedures. In a medium-size center, even five to seven blastocyst thawing procedures per day can be foreseen. However, surprisingly, the studies that have addressed the best timings for blastocyst warming before transfer are limited. Results from these reports are discordant in terms of implantation and pregnancy rates [9,10,11,12], but, in some studies, the number of cycles analyzed was too limited to draw any conclusion [9,10]. Our results support the findings recently published by Ji and coworkers [12] supporting the idea that both post-thaw protocols (short or long culture) can be applied to patients with vitrified day 5 blastocysts.

The interaction between the blastocyst hatching and endometrial receptivity is critical for implantation, although it is challenging to accurately establish the level of synchronicity needed for a successful outcome [3]. To some extent, endometrial receptivity can be managed by artificial endometrial preparation with exogenous hormones. Embryo development in the ART laboratory is, conversely, not very controllable. The possibility to freeze embryos allows providers to overcome the potential asynchrony between endometrium and embryo development, thus optimizing clinical outcomes [2]. Indeed, the developmental delay observed in day 6 frozen–thawed blastocysts does not impact their potential to implant when transferred on the same day as day 5 blastocysts. Similarly, in our approach, blastocysts had always been transferred on the same day as day 5 blastocysts.

On the other hand, we decided to limit this study to blastocysts vitrified on day 5 in order to contain biases related to critical embryological aspects. Indeed, according to some studies, the live birth following frozen–thawed blastocyst transfer was significantly lower with day 6 than with day 5 blastocysts, regardless of their quality [25,26]. Embryo chromosomal abnormalities could conceivably explain the difference in clinical outcomes between the two groups. Indeed, a higher aneuploidy rate has been reported in slower developing blastocysts, with Taylor et al. speculating that the 10% increase in aneuploidy rates in embryos that blastulate on day 6 might be the reason for the lower live birth rate after day 6 thawed blastocyst transfer [27]. Interestingly, Ji et al. demonstrated that the long-culture group was associated with lower implantation, clinical pregnancy and live birth rates and a significantly higher abortion rate following Day 6 blastocyst transfer than those in the short-culture group [13]. Overall, it can be deduced that results obtained evaluating day 5 blastocysts should not be directly applied to day 6 or 7 blastocysts. Further studies are, however, needed to confirm that, to optimize the pregnancy outcomes following transfer of blastocysts vitrified at day 6, a short culture period should be recommended.

In terms of blastocyst quality, we have observed an improvement in the number of top-quality blastocysts following the long culture period. This observation is in line with previous reports [9] and is likely to be explained by a higher rate of re-expansion when increasing the interval between thawing and transfer. As a matter of fact, both less expanded and more expanded blastocysts at the time of vitrification did not seem to be negatively affected by the longer period of culture. A significantly higher clinical pregnancy rate was indeed found in association with the transfer of blastocysts with expansion grade 3 and 4 at vitrification and cultured overnight post-warming. Whether this finding might be as well associated with a higher rate of re-expansion during the longer culture is not clear, since the predictive value of blastocyst re-expansion for the implantation of frozen–thawed blastocysts is still a matter of debate [28].

In regard to obstetric and neonatal outcomes, we did not find any significant differences in terms of spontaneous abortion rate and ectopic pregnancies between the studied groups. Similarly, no differences were found for maternal adverse outcomes and birth weight. Overall, these data appear to indicate that there is no effect of the interval between blastocyst thawing and transfer on these outcomes.

Some strengths and limitations of this study deserve to be commented on. The sample size of this study represents a strength as it allowed us to detect a relative reduction in the live birth rate of at least 20% in association with the long culture period. We could not detect any reduction in ART outcomes following this approach. Moreover, given the use of common protocols for both blastocyst freezing and warming, the generalizability of the results is likely to be solid. Among the limits, we were not able to report the quality of day 3 embryos from which each blastocyst was derived since we commonly use a grouped-embryo culture strategy. However, this parameter does not seem to be critical in the context of this study [29]. As mentioned, the study refers only to day 5 blastocysts, and it refers to data from a single center. Limits also refer to the retrospective nature of the study, so we cannot exclude some allocation biases. As a matter of fact, although the decision to thaw the blastocysts on the day before the transfer was based on the workflow and not on a specific selection, a lower number of top-quality blastocysts at vitrification was found in the analysis in association with the long culture approach (Table 3). On this basis, we can deduce that we may have underestimated the potential benefits of an overnight post-thawing, blastocyst culture before transfer.

## 5. Conclusions

Our results revealed that both short and long blastocyst culture durations guide proper day 5 blastocyst expansion, development, and competence, thus leading to successful and comparable ART outcomes. These results are critical for the organization of workflow in the IVF laboratory. However, randomized trials are required to confirm the present findings.

## Figures and Tables

**Table 1:** Characteristics of the studies evaluating ART outcomes comparing FET cycles divided according to the timing of blastocyst culture before transfer (short, 2–6 h or long, overnight).

Authors, Years	Short Culture			Long Culture			
	***n*** **Blastocysts**	**Day 5** **/Day 6**	**% Live Birth**	***n*** **Blastocysts**	**Day 5/** **Day 6**	**% Live Birth**	***p***
Guerif et al., 2003 [9]	143	Not reported	4	195	Not reported	18	<0.05
Herbemont et al., 2018 [10]	103	Only D5	34.6	115	Only D5	35.8	NS
Haas et al., 2018 [12]	Not assessed	Not assessed	Not assessed	375	Only D5	Not assessed	
Hwang et al., 2020 [11]	56	44/ 11	53.6 *	112	87/ 25	47.3 *	NS
Ji et al., 2022 [13]	1267	486/ 781	Day 5 56.3/ Day 6 48.5	970	483/ 87	Day 5 57.3/ Day 6 35.5	Day 5 NS/ Day 6 < 0.001

NS = not significant; * Ongoing pregnancy rate.

**Table 2:** Basal characteristics and clinical ART indications of the patients who underwent FET cycles divided according to the timing of blastocyst culture before transfer (short, 2–4 h and long, 20–22 h).

Basal Characteristics	Short Culture (*n* = 885)	Long Culture (*n* = 1029)	*p*
Age (years)	36 (33–39)	36 (33–39)	0.29
BMI (Kg/m^2^)	21.5 (19.6–23.9)	21.5 (19.6–24.3)	0.38
AMH (ng/mL)	3.24 (1.79–5.30)	3.16 (1.77–5.01)	0.67
Indication to IVF			0.16
Endometriosis	95 (11%)	106 (10%)	
Unexplained	179 (20%)	169 (16%)	
Tubal factor	130 (15%)	175 (17%)	
Ovulatory disorder	90 (10%)	97 (9%)	
Male factor	271 (31%)	323 (31%)	
Mixed	115 (13%)	157 (15%)	
Others	5 (1%)	2 (0%)	

Data are reported as median (interquartile range) or number (percentage).

**Table 3:** Morphological characteristics of the blastocysts transferred according to the timing of culture before transfer (short, 2–4 h and long, 20–22 h).

Characteristics	Short Culture	Long Culture	*p*
	*n* = 885	*n* = 1029	
TOP quality blastocyst at cryopreservation	478 (54%)	391 (38%)	<0.001
TOP quality blastocyst at transfer	438 (50%)	644 (63%)	<0.001
Expansion grade at vitrification			<0.001
1	28 (3%)	66 (6%)	
2	335 (38%)	549 (54%)	
3	515 (58%)	412 (40%)	
4	7 (1%)	2 (<1%)	
Blastocysts with Expansion grade 1–2 at vitrification			
*n*	363	615	
Clinical pregnancy rate	123 (34%)	234 (38%)	0.22
Live birth	87 (24%)	182 (30%)	0.06
Blastocysts with Expansion grade 3–4 at vitrification			
*n*	522	414	
Clinical pregnancy rate	210 (40%)	197 (48%)	0.03
Live birth	165 (32%)	144 (35%)	0.33

Data are reported as number (percentage).

**Table 4:** Gestational and neonatal outcomes of patients who underwent FET cycles divided according to the timing of blastocyst culture before transfer (short, 2–4 h and long, 20–22 h).

Pregnancy/Neonatal Outcomes	Short Culture (*n* = 885)	Long Culture (*n* = 1029)	*p*
Spontaneus Abortion ^a^	72 (22%)	95 (22%)	0.93
Ectopic pregnancy ^a^	2 (1%)	7 (2%)	0.31
Intrauterine Fetal Death ^a^	0 (0%)	1 (<1%)	1.00
Gestational Diabetes ^b^	17 (7%)	13 (4%)	0.19
Hypertension ^b^	6 (2%)	3 (1%)	0.19
Placenta Previa ^b^	8 (3%)	6 (2%)	0.41
Preterm labor ^b^	14 (6%)	14 (4%)	0.56
Low birth weight (<2500 g) ^b^	18 (7%)	19 (6%)	0.61
Total gestational issues	47 (19%)	44 (14%)	0.11

Data are reported as number (percentage). ^a^ Data are calculated on total number of pregnancies (Short culture: *n* = 333; Long culture: *n* = 431); ^b^ Data are calculated on total number of live births (Short culture: *n* = 252; Long culture: *n* = 326).

## Data Availability

Not applicable.
